# Recognition of Prodromal Hypoglossal Nerve Palsy Presenting with Neck Pain as Primary Complaint: Findings from a Rare Case Report in Direct Access Physiotherapy during the COVID-19 Pandemic

**DOI:** 10.3390/healthcare11091342

**Published:** 2023-05-07

**Authors:** Firas Mourad, Claudia Milella, Graziana Lullo, Francesco Zangari, Roberto Meroni, Alan Taylor, Roger Kerry, Nathan Hutting, Filippo Maselli

**Affiliations:** 1 Department of Physiotherapy, LUNEX International University of Health, Exercise and Sports, 4671 Differdange, Luxembourg; 2 Luxembourg Health & Sport Sciences Research Institute A.s.b.l., 50, Avenue du Parc des Sports, 4671 Differdange, Luxembourg; 3 Department of Human Neurosciences, Sapienza University of Rome, 00185 Rome, Italy; 4 Radiology Service, Azienda Socio Sanitaria Territoriale della Franciacorta, Viale Mazzini 4, 25032 Chiari, Italy; 5 Faculty of Medicine and Health Sciences, School of Health Sciences, Division of Physical Therapy and Sport Rehabilitation, University of Nottingham, Nottingham NG5 1PB, UK; 6 Department of Occupation and Health, School of Organisation and Development, HAN University of Applied Sciences, 6503 GL Nijmegen, The Netherlands

**Keywords:** cranial nerves, differential diagnosis, rehabilitation, red flags, direct access

## Abstract

Neck pain (NP) is the second most common musculoskeletal disorder. Spinal cysts (SCs) are cystic dilatations of the synovial sheaths in joints and tendons. SCs are extremely rare in the cervical spine. Typically, patients are unaware of having an SC due to its asymptomatic nature; however, when cervical SC extends, its volume could compress the surrounding structures, such as the hypoglossal nerve. Isolated hypoglossal nerve palsy (HNP) is very rare and typically presents with unilateral atrophy of the musculature of the tongue and contralateral tongue deviation. Often, patients with HNP also report occipital/neck pain. A 75-year-old man with occipital/neck pain as a primary complaint. Although difficult to observe because of the filtering facepiece two mask, difficulties in articulation and sialorrhea during the interview were noticed. These latter were cues to consider CN examination that revealed CN XII palsy. This prompted a referral for further examination that revealed an SC compressing the right hypoglossal canal. The patient was not considered a surgical candidate and was managed conservatively. This case report outlines the relevant findings relating to the triage of a rare isolated hypoglossal nerve palsy from the physiotherapist’s perspective within a complex setting because of the COVID-19 pandemic. Although referred with a diagnosis of cervical radiculopathy, our case highlights that skilled physiotherapists may play a fundamental role in both the recognition and, when applicable, subsequent novel management of a non-musculoskeletal presentation.

## 1. Introduction

Neck pain (NP) is the second most common musculoskeletal disorder being the fourth leading cause of disability, with an annual prevalence rate exceeding 30% [1,2]. In 2017, the Global Burden of Disease reported that both the incidence and prevalence of NP are increasing with age and are greater among females [2]. The physical, psychological, and socioeconomic impact of NP is relevant [1], representing 25% of the workload of outpatient physiotherapy [3]. Additionally, general practitioners are consulted seven times per week for NP [4]. Notably, the number of people who present for a physiotherapy consultation with a specific condition masquerading as musculoskeletal NP is unknown [5,6,7,8,9,10]. Although the incidence of serious pathology of the neck is low, it represents up to 2% of the referred patients [2].

Clinical practice guidelines recommend ruling out signs or symptoms of major structural pathologies (namely, NAD IV)—such as congenital craniovertebral anomalies, blood flow limitation, anatomical instabilities, and autonomic disorders—masquerading NP before providing any evidence-based intervention [3,11,12,13]. Potentially, serious pathologies of the neck can result in cranial nerve (CN) palsy [7,9,14,15]. However, the detection of pathological conditions of cranial nerves represents a real challenge for clinicians since most patients do not present with specific signs and symptoms limited to the territory of one particular nerve [16,17].In addition, CN palsies typically manifest as transient neurological signs and symptoms, and often patients do not mention them spontaneously during the consultation because they are vague and subtle in their early manifestation [18]. Those signs and symptoms are identifiable during the patient’s history with specific questioning or noticed during visual face inspection, allowing the clinician to support or not a medical referral [5,7,18]. Notably, wearing a filtering facepiece (FFP) mask due to the COVID-19 pandemic increases the risk of losing important cues during the encounter of a person with NP, headache, and/or orofacial symptoms with potential CN involvement, increasing the risk of delayed diagnosis of serious pathologies, which already possess a high (i.e., 5–20%) incidence [9,19,20,21]. Therefore, it is the responsibility of all physiotherapists to be capable and skilled in identifying these patients during the subjective history and further verifying during the physical examination [6,7,8,9,10,22].

The hypoglossal nerve constitutes the XII pairs of the CNs. Hypoglossal nerve palsy (HNP) is a common finding in neurological diseases, and it’s often associated with other cranial nerve palsies (i.e., glossopharyngeal, vagal, and spinal accessory CNs) [23,24,25,26,27,28,29,30,31]. That is, the etiology of HNP includes: primary or metastatic tumor, followed by trauma to the face and/or neck (i.e., occipital condyle fractures), stroke, infection, radiation, skull base disorders, craniocervical degenerative disease (i.e., cervical osteophytes, arachnoid cysts, juxta facet synovial cyst), Eustachian tube dysfunction or disorders, inflammatory diseases (i.e., Wegener’s granulomatosis or rheumatoid arthritis), upper and lower motor neuron disease (i.e., multiple sclerosis, Gullain-Barrè) and iatrogenic injury (i.e., hypoglossal neurapraxia after airway extubation) [23,24,25,26,27,28,29,30,31]. Although the pathogenesis is controversial, an association between HNP and viral infection—such as infectious mononucleosis, common cold, and influenza vaccination—has been reported, and only 3% of HNP are considered idiopathic [24,32,33,34,35]. Intrinsic risk factors for HNP are related to the anatomical location of the nerve, including calcified styloid ligament, instability, or bony abnormalities of the craniovertebral junction, and short neck [31].

The clinical presentation of HNP is often variable and depends on the location and cause of the paralysis: an incomplete history and physical examination may lead to a missed diagnosis [7]. Isolated HNP is very rare and typically presents with unilateral atrophy of the musculature of the tongue and contralateral tongue deviation [32]. Other common symptoms are dysphagia, dysarthria, hypophonia, tongue weakness, difficulty in mastication, swallowing solid food, and drinking [33,36]. Interestingly, 42% of these patients also report occipital pain, typically described as neuralgiform [33,36].

The purpose of our case report is to discuss relevant aspects of pathophysiology and the differential diagnosis of a rare isolated HNP of a patient referred to physiotherapy by a medical doctor with the diagnosis of cervical radiculopathy during the COVID pandemic. Physiotherapists who manage persons presenting with neck, head, and/or orofacial pain and associated symptoms should be trained to recognize prodromes and alarming signs and symptoms to consider testing CNs [37,38,39]. That is, gaining a working knowledge of the individual CN function and dysfunction allowed the physiotherapist to make sense of a potentially confounding scenario due to the inability to observe the patient’s face because of the FFP2 mask and then filter out a patient in need of a referral. To the best of the author’s knowledge, this is the first case report describing an isolated HNP recognized by physiotherapists after a medical doctor referral. Our case report was designed following the CARE guidelines [40].

## 2. Case Presentation

**Figure 1:**
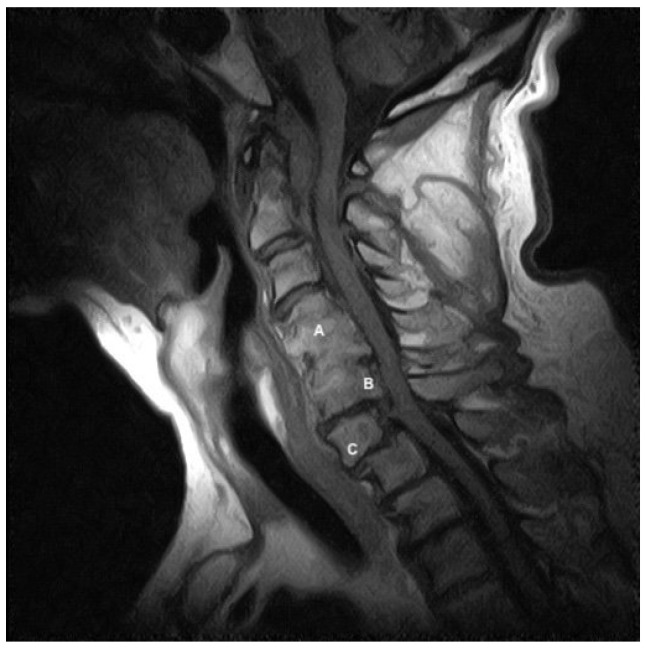
Sagittal T1-weighted MRI image reveals a synostosis at C4-5 (A), a retrolisthesis of C6-7 (B), and an anterolisthesis of C7-T1 (C).

A 75-year-old male attended our physiotherapy clinic seeking help for occipital/neck pain. The symptoms started one year prior to the physiotherapy consultation, and at the time, were intermittent, with a 5/10 NPRS resting pain. The pain was worst during the morning and at night-time when lying in bed for sleeping. He was used to doing Pilates and swimming regularly. However, the subject was forced to dismiss any activity because of the pandemic restrictions, having a progressive worsening in the intensity and frequency of his neck pain. Recently, the subject had a left total hip arthroplasty well recovered after a short period of rehabilitation. After surgery, he noticed a progressive worsening of his NP, having his worst pain with 7/10 NPRS and noticeable difficulties in active cervical rotational movements. He also noticed a new spontaneous intermittent sharp pain just below the base of the skull in the upper cervical spine, most noticeable with right rotational movements, which were the most restricted movements. He reported a recent onset—2 months ago—of paresthesia on the fifth finger of his right hand, progressively changing into markable hypo-aesthesia. His general practitioner suggested radiographic scans without physically visiting him as a preauction for the COVID-19 pandemic (i.e., telehealth). Imaging revealed a synostosis at C4-5, a retrolisthesis of C6-7, and an anterolisthesis of C7-T1 (Figure 1. Note that radiography was not available during the physiotherapy consultations, and the physiotherapist did not feel relevant to having a view after the visit, as described in the following paragraphs. However, for clarity and transparent reporting, the authors felt it relevant to report the finding with an MRI image); thus, the general practitioner suggested physiotherapy and some painkiller for suspected cervical radiculopathy in a follow-up consultation. The subject did report familiarity with rheumatoid arthritis by his mother’s family side (i.e., aunt and mother). No other red flags, trauma, head/neck injury, or significant concomitant pathologies were reported. The main reasons for the patient to seek physiotherapy care were to receive explanations of his symptoms, to receive treatment for pain reduction, to reduce movement deficits, and advice for neck exercises and self-management strategies.

## 3. Physical Examination

**Figure 2:**
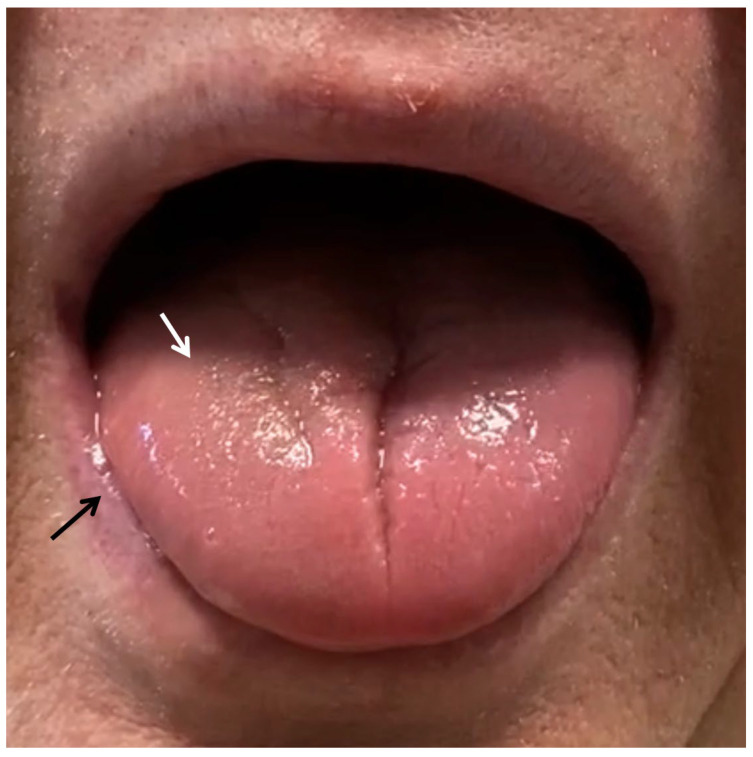
Hypoglossal nerve examination. Right-sided hypotrophy and reduced tone of the tongue on the right affected side (white arrow) during the inspection. Notice also sialorrhea highlighted by the black arrow.

A revision of the systems was performed with the goal of excluding any specific pathologies underlying the clinical presentation. His wrist, hand, and finger presented restriction during passive mobilization bilaterally. Although rheumatoid arthritis was never diagnosed, he presented symmetrical finger swelling and deformation on both hands. Additionally, he presented atrophy on his right thenar eminence. Accordingly, a peripheral neurological examination was performed: pinprick, cold, and light touch sensibility were reduced on the ulnar territory of his right hand; in addition, a reduction in force for the first digit adduction and finger extension was observed. Upper limb neurodynamic test (ULNT) 1 testing was unremarkable because of the extension mobility reduction of both wrists. Palpation of the nerve root at the scalene muscle reproduced pain on the neck and shoulder, and by the time his familiar paresthesia. His neck’s active right rotation was 50 degrees, with a sharp pain at the base of the skull at the end of the permitted right rotation. Although difficult to observe because of the FFP2 mask, difficulties in articulation and sialorrhea during the interview were noticed. These latter were cues to consider CNs examination: particularly, CN XII testing revealed atrophy and asymmetrical deviation of the tongue toward the affected side (i.e., the right) (Figure 2 and Video S1). Drooling was also observed (i.e., decreased clearance of saliva) and power reduction of the tongue when tested against the physiotherapist’s resistance outside the check while the patient was pushing out his cheek with the tongue (Video S2). No further examination was performed. We recommend readers integrate this case with the article of Taylor et al. [7] for further details on a function-based approach to cranial nerve testing and Mourad et al. [9] for a screening for referral decision tool.

Video S1. CN XII testing revealed asymmetrical deviation of the tongue toward the affected side (i.e., right side). 

Video S2. CN XII testing revealed power reduction of the tongue when tested against the physiotherapist’s resistance outside the check while the patient was pushing out his cheek with the tongue. Notice the power reduction toward the opposite side (i.e., the left side) and the increased force needed by the physiotherapist to resist when assessing the deviation toward the right. The genioglossus muscles receive motor innervation by the ipsilateral hypoglossal nerve, and its contraction provides tongue deviation toward the opposite side. Lower motor neuron palsy (i.e., at the level or below the level of the nucleus) of the hypoglossal nerve determines contralateral deviation power reduction of the tongue, resulting in a tongue movement toward the palsy side. Notice also the need for the patient to remove the excess saliva in the mouth beyond the lip at the end of the test.

## 4. Analysis

Because the patient’s presentation was suggestive of CN palsy, the decision was made that he was in need of a referral to a neurosurgeon. Although the patient was wearing an FFP2 mask due to the COVID-19 pandemic, and even if the patient did not think to mention it spontaneously during the history taking, difficulties in articulation and sialorrhea during the interview were noticed. After noticing it, the patient reported having also experienced also recent speech (i.e., articulation) difficulties and drooling, which were a cue to consider testing the CNs, revealing a hypoglossal nerve palsy. That is, it is critical that clinicians ask questions for the recognition of transient or subtle antecedent neurological signs and symptoms and to be able to interpret the complex presentations that commonly combine neck pain with associated disorders [5,6,7,8,18,41]. The process of interpreting the data from the patient history and defining the patient’s risk profile is essential to an effective evaluation, including the physical examination: CN examination should be an integral part of that process, having the objective of triaging those patients in need for further examination or referral [5,6,7,8,18,41]. In our case, the CN examination allowed the physiotherapists to offer a much clearer referral, making a recommendation for further examination and which offered both a reported suspicion of compressive palsy and an anatomical clue to the potential pathology [5,6,7,8,18,41]. That is, the sound knowledge of the dysfunction and the recognition of pathological patterns allowed the clinician to dispositionally weigh subtle cues gained in a complex and confounding scenario (i.e., the FFP2 mask due to the COVID pandemic situation and the unreported neurological signs) and that lead to a targeted physical examination [5,6,7,8,18,41].

The hypoglossal nerve is a purely motor nerve, and its composition is very complex. It is the 12th cranial nerve, and it’s the most medial of the lower cranial nerves [17]. The hypoglossal nerve is divided into five segments: the first or medullary segment, the second or cisternal segment, the third or skull base segment, the fourth or nasopharyngeal carotid segment, and the fifth or sublingual segment. It arises from the hypoglossal nucleus in the medulla and emerges as multiple radicles between the pyramid and olive [42]. The nerve passes anterolaterally into the posterior cranial fossa and through the hypoglossal canal into the cranium. The occipital condyles (in their inferior portion), the jugular foramen, the jugular process of the occipital bone, and the sphenoid part of the clivus comprise the hypoglossal canal [17,42]. After passing through the canal, the hypoglossal nerve exits the neurocranium. Then, it passes inferiorly in the neck, under the sternocleidomastoid muscle, over the internal carotid artery, and medially to the 9th, 10th, and 11th cranial nerves. The nerve travels deep to the posterior belly of the digastric muscle and crosses the lateral surface of the external carotid artery, reaching the anterior border of the sternocleidomastoid [42]. The hypoglossal nerve usually travels inferiorly to the Wharton duct and lingual artery and ends by innervating the tongue musculature, particularly the extrinsic and intrinsic muscles of the tongue, except the palatoglossus muscle, which is supplied by the vagus nerve. At this level, the nerve joins general somatic efferent fibers from the first cervical nerve C1 [42]. The latter courses with the hypoglossal nerve for a short distance and then drifts away to innervate the geniohyoid, the thyrohyoid muscles, as well as the infrahyoid muscles. The hypoglossal nerve, throughout its course, receives fibers from the nodose ganglion of the 10th cranial nerve at the level of the hypoglossal canal and postganglionic sympathetic fibers from the superior cervical ganglion at the level of the atlas [42]. It also communicates with the lingual branch of the mandibular nerve at the base of the tongue. The sternocleidomastoid muscle can be additionally innervated from the C1 nerve traveling in conjunction with the hypoglossal nerve [42]. Additionally, a combined articular branch derived from a plexus formed by the hypoglossal and C1 nerves entering the atlanto-occipital joint on its lateral surface just superior to the transverse process of the atlas. The hypoglossal nerve regulates all tongue activity and its movement [42].

## 5. Follow-Up and Outcomes

**Figure 3:**
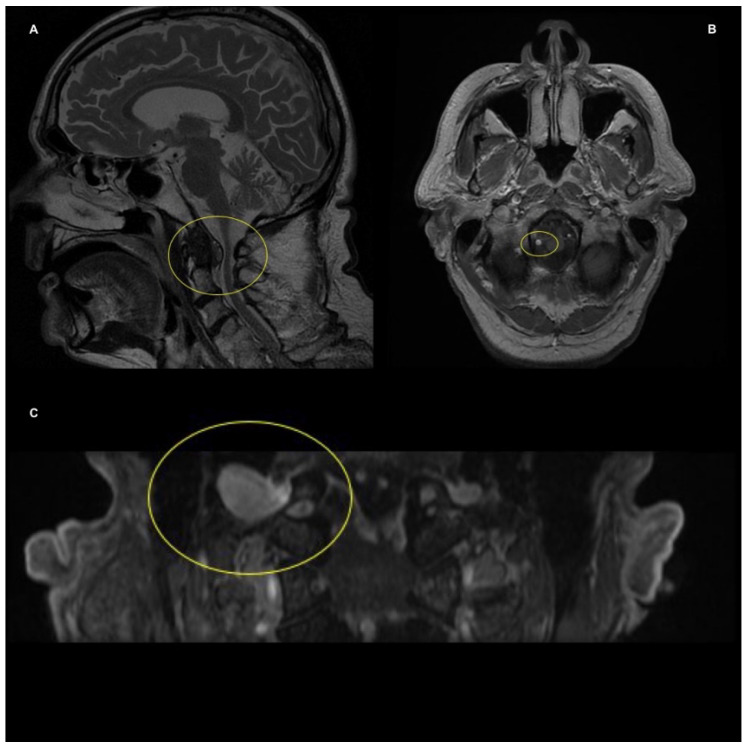
(**A**). Sagittal T2-weighted MRI image details a hyperintense synovial cyst adjacent to the right side of the craniocervical joint; (**B**). Axial T1-weighted image shows a hypointense mass in the posterior cranial fossa; (**C**). Coronal T1-weighted MRI image provides references on the size of the cyst.

The neurosurgeon suggested a magnetic resonance imaging (MRI) scan with contrast to the craniocervical region. The MRI revealed a 9 × 12 × 15 mm hypointense mass in the posterior cranial fossa on T1-weighted images; the T2-weighted images detailed a hyperintense synovial cyst (SC) adjacent to the right side of the craniocervical joint, that was compressing the right hypoglossal canal (Figure 3).

**Figure 4:**
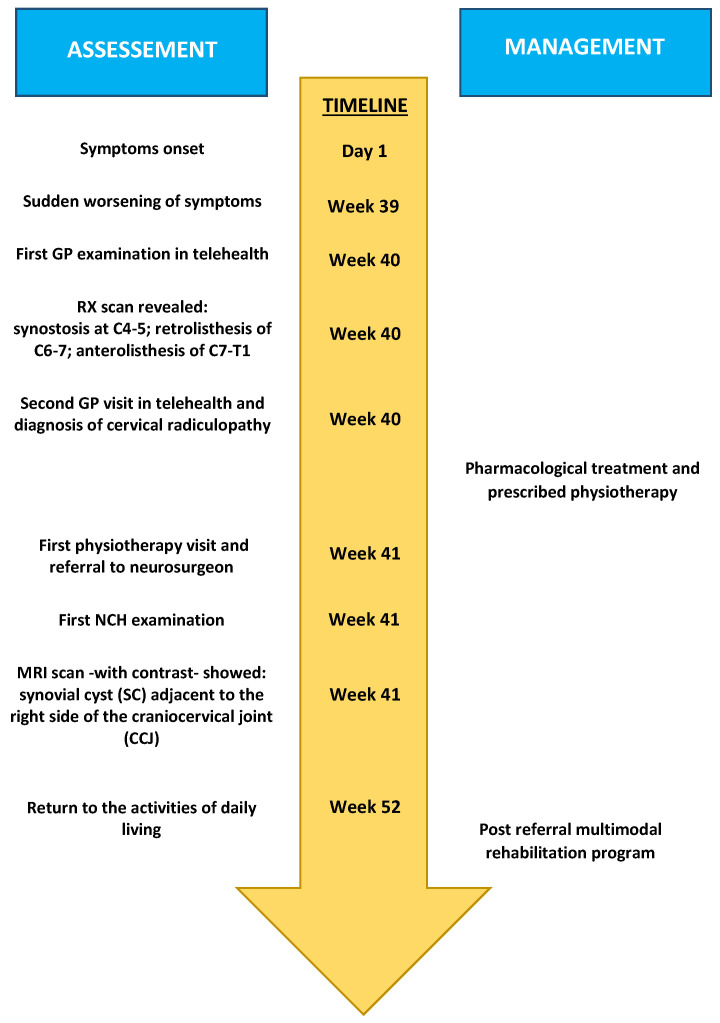
Timeline.

The delayed diagnosis of SC may have been due to the inadequate evaluation of imaging (i.e., radiography), not only inadequate examination due to telehealth, as MRI evaluation, including T2WI modality, is warranted to detect SC. The neurosurgeon felt that the patient was not a surgical candidate for the SC removal (i.e., risk-benefit ratio), and a cautious physiotherapy program was advised for the patient’s cervical and radicular symptoms. For a detailed description of the timeline management, see Figure 4.

The patient was treated one time per week for three months with a multimodal program consisting of gentle manual therapy to the neck (i.e., traction, mobilization, and mobilization with movement), neurodynamic to the affected upper limb (ULNT 1 and 3 sliders and tensioner), electric dry needling to the upper cervical muscles and the neural tissue of the affected upper limb, with the goal to modulate symptoms. Mobility and strengthening exercises to the cervical and scapulothoracic region with progressive load were prescribed; also, specific exercises for the recovery of the function of the oro-facial-buccal muscles, of the articulation, and of the tongue force were also delivered. At the follow-up, the subject’s pain resolved over time with an improvement of his right neck rotation (i.e., 70 degrees), and his peripheral neurological symptoms got better; however, although his speech and sialorrhea improved over time, he continued to present deviation and power reduction of the tongue. The capability of the physiotherapist to appropriately refer the patient and the gentle progressive management strategies were a strength to the therapeutic alliance, leading the patient to fully adhere to and tolerate the treatment plan. Although the aim of the present case is to describe the physiotherapist’s screening for the referral process, we invite readers to refer to Table 1 for more details related to the treatments. 

## 6. Discussion

Red flags are signs and symptoms that should alert physiotherapists to consider carefully if the patient is within the scope of practice or whether an appropriate medical referral is needed [5,6,7,8,9,43,44]. Commonly, patients with serious pathology of the neck present subtle transient antecedent neurological signs or symptoms (e.g., acute onset of unusual headache or NP, and/or ischemic signs and symptoms) or risk factor (e.g., recent head/neck trauma) [5,6,7,8,9,43,44]. An increasing body of evidence suggests neurological examination (i.e., peripheral nerves, CNs, and upper motor neuron testing) as an important competence for physiotherapists when examining for potentially serious conditions of the neck [5,6,7,8,9,43,44]. Typically, due to their anatomical location, the inferior portion of CNs (i.e., CN V trigeminal, CN VII facial, CN IX glossopharyngeal, and CN X vagus nerves, CN XII hypoglossal) and the nerves of the superior cervical spine (e.g., the occipital nerve) are involved in facial syndromes and NP [9]. That is, the peripheral extraforaminal segments of these nerves travel below the base of the skull [17]; thus, their involvement should be considered in cases of upper neck pain/headache, neuralgic pain, disturbed speech, swallowing, coughing, deglutition, sensory dysfunctions, taste, autonomic dysfunctions, dysphagia, pharyngeal pain, cardiac or gastrointestinal compromise, and weakness of the trapezius, of the sternocleidomastoid, or of the tongue muscles [7,42].

In our case, the NP associated with the onset of hypo-aesthesia on the fifth finger of the patient’s right hand were the main symptoms that led the medical doctor to refer the subject for physiotherapy. The complaint of a new spontaneous intermittent sharp pain just below the base of the skull, the rapid increase in the intensity of neck pain, the marked reduction in right rotational ROM, the difficulties in speaking, and the sialorrhea led the dispositional clinical reasoning of the physiotherapist to the decision that the patient’s condition required a referral to a neurosurgeon [5,7,14,15]. The following MRI investigation detected an atlantooccipital SC, which was compressing the hypoglossal nerve along its canal, causing an isolated HNP. MRI with or without contrast of the skull base and brain focusing the hypoglossal nerve and its course, and MRI neurography, an evolution of conventional MRI, should be used while evaluating the cause of suspected HNP [17,28]. In addition, high-resolution basicranial computerized tomography (CT) scans may be considered when evaluating osseous involvement, such as degenerative osteoarticular diseases or craniovertebral junction trauma [17,28].

Spinal SC is extremely rare in the cervical spine and manifests during the fifth decade with no gender predominance [45]. SC are cystic dilatations of the synovial sheaths that are often found in joints and tendon sheaths. The etiology of spinal SC is unclear, and many theories have been proposed to explain their pathogenesis [45,46]. Possible causes include synovial fluid extrusion from the joint capsule, increased motion at the spinal joint, myxoid degeneration, cyst formation in the synovial tissue, and latent growth of developmental rests of synovial tissue or tissue metaplasia [47]. These cysts, when degenerative, result from an extrusion of the synovium through a capsular because of a degenerated or unstable facet joint [47]. SCs in the neck involve both the atlanto-odontoid process articulation (with or without atlanto-axial dislocation), C1-C2 zygapophyseal articulations, and, more rarely, the atlanto-occipital joints [45]. Typically, patients are unaware of having an SC due to its asymptomatic nature; however, when an SC extends its volume and invades and compresses the surrounding structures, it may cause nerve root or spinal cord compression resulting in pain and/or neurological symptoms [48]. In our case report, a 75-year-old male with an atlantoaxial SC had symptoms related to the isolated compression of the right hypoglossal nerve related to the close anatomical relationship between the hypoglossal nerve and the atlanto-occipital joint.

The management of the SC is still under debate, and several treatments have been suggested, including surgical resection, CT-guided cyst surgical aspiration, or steroid facet injection. However, a conservative approach using collars [49,50,51] or external spine immobilization has been shown to have benefits for cyst regression [45]. Notably, when the SC is located at the cervical level and presents the potential to compress any vital structures, surgery is the treatment of choice [45]; the approach choice depends on the surgeon’s preference and on the extent of the SC [47,52]. Lateral suboccipital craniectomy or juxta condylar approaches are the most described surgical procedures. Generally, cysts located at different sites may resolve spontaneously with clinical improvement; however, data on craniocervical SC behavior and spontaneous regression are not available. To the best of our knowledge, only one case report described a spontaneous SC resolution associated with neurological symptom improvement [45].

To the best of the authors’ knowledge, this is the first case report that describes the clinical reasoning and the decision-making process that led a physiotherapist to refer a patient with SC causing an isolated HNP presenting neck/occipital pain. However, our article presents a single episode of care and may represent an outlier in clinical practice, necessitating caution regarding generalizability. In addition, a multimodal management approach consisting of manual therapy and specific exercises directed to the tongue, the neck, and shoulders was never described before for the treatment of HNP. The benefits provided by physiotherapy may rely on the neuroanatomical and functional relationship between the cervical spine and the stomatognathic system; that is, alterations in the lips, the tongue, the jaw, the cheeks, and the stomatognathic functions have been already described in the literature as orofacial myofunctional disorders (OMDs) [53]. OMDs can impair both the cervical and the temporomandibular function being reciprocally contributing factors [54]. In our case, HNP secondary to an SC compression at the atlanto-occipital joint compromised the patient’s orofacial function, potentially triggering NP by the convergence of cervical nociceptive afferents at the level of the cervical trigeminal nucleus. The afferents fibers from the lingual nerve (trigeminal and hypoglossal anastomosis) traveling to the upper cervical ventral ramus provide a plausible explanation for both neck symptoms and the maladaptive response of the orofacial musculature to the impaired function on the tongue [55,56]. All of the above lead the authors to believe that the present case may represent a novel approach for the management of isolated HNP.

## 7. Conclusions

Neck pain is commonly encountered in physiotherapy outpatient settings, and delayed diagnosis of serious presentation masquerading musculoskeletal NP is frequent. With physiotherapists assuming the responsibility of the first contact role, it is essential that physiotherapists possess the triage skills (namely, screening for referral) to recognize the key elements of the patient history and the physical examination which may be indicative of a condition outside the scope of practice. This case report outlines the relevant aspects relating to the pathophysiology, screening, and differential diagnosis of an isolated hypoglossal nerve palsy secondary to degenerative changes of the cervical spine. It also describes the relevant findings from the history and physical examination from the physiotherapist’s perspective of an old patient with a rare condition within a complex and confounding scenario. Although the patient was referred by a physician with a diagnosis of cervical radiculopathy, our case highlights that skilled physiotherapists may play a fundamental role in both the recognition and subsequent novel management of a non-musculoskeletal presentation.

## Figures and Tables

**Table 1:** Summary of the detailed management plan. ROM, range of motion; NPRS, numeric pain rating scale.

Intervention	Rationale	Dosage/Progression	Baseline Outcomes	Discharge Outcomes
Manual Therapy	To increase ROM and decrease pain	Traction (sessions 1 to 4) Mobilization (sessions 5 to 8) Mobilization with movement (sessions 9 to 12)	5-7/10 NPRS resting pain 45 degrees cervical right rotation ROM	No pain 70 degrees cervical right rotation ROM
Upper Limb Neurodynamic sliders and tensioner	To reduce the neural mechanosensitivity	Gentle sliders for the median and ulnar nerves (sessions 1 to 4) End-range sliders for the median and ulnar nerves (sessions 5 to 8) Tensioner for the median and ulnar nerves (sessions 9 to 12)	Nerves spontaneous pain at rest and reduced excursion of the nerve during upper limb neurodynamic techniques	Neuropathic pain solved and full excursion of the nerve during upper limb neurodynamic techniques
Electric dry needling	Reduce muscle spasm and tissue irritability	Directed to upper cervical muscles for the first 2 sessions and along the neural tissue (i.e., perineural) of the affected upper limb at the begging of sessions 3 and 4.	5-7/10 NPRS resting pain 45 degrees cervical right rotation ROM	No pain 70 degrees cervical right rotation ROM
Exercise therapy	To improve function, pain control, and disability reduction	Mobility and strengthening exercises to the cervical and scapulothoracic region with progressive load (e.g., gentle unloaded craniocervical flexion to isometric head raise). Repetition and sets followed a low load regimen (e.g., 3–6 repetitions for 1–2 sets) and were prescribed based on the patient’s tolerance.	55 degrees active flexion-extension cadence (measured by an inclinometer) 115 degrees active rotational cadence (measured by an inclinometer) Neck Disability Index = 60% (severe disability)	80 degrees active flexion-extension cadence (measured by an inclinometer) 140 degrees active rotational cadence (measured by an inclinometer) Neck Disability Index = 20% (mild disability)
Specific exercise therapy	To improve oro-facial-buccal functions, including the tongue	Frequent daily home exercise program focused on tongue function (e.g., isometric strengthening exercises of the tongue, especially in abduction, and articulation exercises to lips, tongue, and mouth to improve physical function)	Very low tongue abduction power (i.e., not able to overcome small resistance) measured by the physiotherapy hand resistance Subjective perception of the amount of drooling measured by the frequency of lips clearance = “very frequent”	Moderate tongue abduction power (i.e., able to overcome a small/moderate resistance isometrically) measured by the physiotherapy hand resistance Subjective perception of the amount of drooling measured by the frequency of lips clearance = “50% less frequent”

## Data Availability

Not applicable.

## Supplementary Materials

The following supporting information can be downloaded at: https://www.mdpi.com/article/10.3390/healthcare11091342/s1. Video S1. CN XII testing revealed asymmetrical deviation of the tongue toward the affected side (i.e., right side). Video S2. CN XII testing revealed power reduction of the tongue when tested against the physiotherapist’s resistance outside the check while the patient was pushing out his cheek with the tongue.
